# Distinct Regulation of Host Responses by ERK and JNK MAP Kinases in Swine Macrophages Infected with Pandemic (H1N1) 2009 Influenza Virus

**DOI:** 10.1371/journal.pone.0030328

**Published:** 2012-01-18

**Authors:** Wei Gao, Wenkui Sun, Bingqian Qu, Carol J. Cardona, Kira Powell, Marta Wegner, Yi Shi, Zheng Xing

**Affiliations:** 1 Medical School and the State Key Laboratory of Pharmaceutical Biotechnology, Nanjing University, Nanjing, China; 2 Department of Respiratory Medicine, Clinical School of Medicine of Nanjing University, Nanjing General Hospital of Nanjing Military Command, Nanjing, China; 3 Department of Respiratory Medicine, the Second Military Medical University, Nanjing General Hospital of Nanjing Military Command, Nanjing, China; 4 Department of Veterinary Biomedical Sciences, College of Veterinary Medicine, University of Minnesota at Twin Cities, Saint Paul, Minnesota, United States of America; University of Georgia, United States of America

## Abstract

Swine influenza is an acute respiratory disease in pigs caused by swine influenza virus (SIV). Highly virulent SIV strains cause mortality of up to 10%. Importantly, pigs have long been considered “mixing vessels” that generate novel influenza viruses with pandemic potential, a constant threat to public health. Since its emergence in 2009 and subsequent pandemic spread, the pandemic (H1N1) 2009 (H1N1pdm) has been detected in pig farms, creating the risk of generating new reassortants and their possible infection of humans. Pathogenesis in SIV or H1N1pdm-infected pigs remains poorly characterized. Proinflammatory and antiviral cytokine responses are considered correlated with the intensity of clinical signs, and swine macrophages are found to be indispensible in effective clearance of SIV from pig lungs. In this study, we report a unique pattern of cytokine responses in swine macrophages infected with H1N1pdm. The roles of mitogen-activated protein (MAP) kinases in the regulation of the host responses were examined. We found that proinflammatory cytokines IL-6, IL-8, IL-10, and TNF-α were significantly induced and their induction was ERK1/2-dependent. IFN-β and IFN-inducible antiviral Mx and 2′5′-OAS were sharply induced, but the inductions were effectively abolished when ERK1/2 was inhibited. Induction of CCL5 (RANTES) was completely inhibited by inhibitors of ERK1/2 and JNK1/2, which appeared also to regulate FasL and TNF-α, critical for apoptosis in pig macrophages. We found that NFκB was activated in H1N1pdm-infected cells, but the activation was suppressed when ERK1/2 was inhibited, indicating there is cross-talk between MAP kinase and NFκB responses in pig macrophages. Our data suggest that MAP kinase may activate NFκB through the induction of RIG-1, which leads to the induction of IFN-β in swine macrophages. Understanding host responses and their underlying mechanisms may help identify venues for effective control of SIV and assist in prevention of future influenza pandemics.

## Introduction

Swine influenza is an acute respiratory disease caused by swine influenza viruses (SIV). The symptoms and signs generally include fever, sneezing, nasal rattles, and respiratory distress in pigs. Pigs recover within a few days, but severe signs can develop and mortality can reach up to 10% when highly virulent strains are involved [1] or pigs are infected at young ages [2], [3]. Pigs have long been considered to be the intermediate host of various subtype viruses and “mixing vessels” for the evolution and genesis of influenza viruses with pandemic potential because of their susceptibility to swine, avian, and human influenza viruses [4], [5], [6]. This broad susceptibility is due to the presence of both sialic acid (SA)2,3 Gal- and SA2,6-Gal receptors present in the respiratory epithelium.

Three major SIV subtypes are prevalent: H1N1 (classical swine H1N1 and avian-like H1N1), H3N2 (triple reassortant H3N2 and human-like H3N2), and H1N2 [2], [7], [8], [9], [10], [11]. Pigs are also susceptible to and show clinical signs when infected with pandemic (H1N1) 2009 virus (referred to hereafter as H1N1pdm) [12], which emerged in April 2009 in North America [13], arising at least in part from contemporaneous SIV. To date H1N1pdm has been found in a few swine farms [12], [14], [15], which further demonstrates a two-way process of both gene and virus trafficking between humans and pigs. Though H1N1pdm has remained antigenically and genetically stable in humans since its emergence, a novel reassortant SIV containing a H1N1pdm-like NA and seven other genes from triple-reassortant H1N2 and European “avian-like” H1N1 viruses was identified in early 2010 [16], and that same year H1N1pdm was shown to be evolving genetically at a faster pace in pigs than it was in humans [12], [15], [17]. Effective control of circulating influenza viruses in swine populations is key to reducing consequent genesis of novel pandemic strains that threaten the health of both humans and animals.

Studies have been conducted to identify proinflammatory cytokines including TNF-α, IL-6, IL-12, and IFN-α or IFN-γ, which are upregulated in lung or bronchoalveolar secretions in SIV-infected pigs [18], [19], [20], [21] and may be correlated with clinical manifestations. In an alveoli macrophage-depleted pig model, macrophages appeared to be indispensible to effective clearance of SIV from lungs. A higher frequency of cytotoxic T, γδ T, and Treg cells were also detected in infected pig lungs [18], which together with the induction of cytokines, contribute to pathogenesis of influenza infection in pigs. Exploring the mechanism of regulation of host responses is crucial for understanding the pathogenesis of SIV and for controlling swine influenza in pigs. Macrophages residing beneath the respiratory epithelium and surrounding alveoli are part of the first line defenses against influenza viruses. During influenza viral replication in bronchial epithelial cells, macrophages are one of the earliest targets to be infected. Together with dendritic cells, macrophages coordinate innate immune responses, which subsequently lead to adaptive immunity by initiating antigen presentation and lymphocyte activation. Macrophages are indispensable in alveolar host defense and controlling influenza virus in pulmonary organs in pigs [22]. While protective in launching host antiviral responses and restricting virus spread, induced proinflammatory cytokines and chemokines are also the cause of pathogenicity for the host and may lead to acute respiratory failure (ARF), a major cause of death in highly pathogenic H5N1 or H1N1pdm-infected humans [23]. Needless to say, the roles of macrophages are critical to pathogenicity as well as host protection in SIV-infected pigs. However, little is known about the mechanisms of how host responses are regulated in pigs or their macrophages.

Considering the critical role macrophages play in SIV infections, and the threat that H1N1pdm could further evolve higher virulence in pigs and subsequently infect humans, we were interested in profiling host responses of swine macrophages to H1N1pdm, and more importantly, in exploring the underlying mechanism of host response regulation including antiviral, proinflammatory responses, and apoptosis in pigs. In this report, we will demonstrate that swine macrophages are susceptible to infection by H1N1pdm. We will show a unique pattern of proinflammatory cytokine responses to the infection, which are distinctly regulated by swine mitogen-activated protein (MAP) kinases. We have also observed cross-talk between MAP kinase and NFκB pathways, and our data indicate that MAP kinase ERK1/2 and JNK1/2 may impact the activation of NFκB through the induction of RIG-1, leading to IFN-β induction in H1N1pdm-infected swine macrophages.

## Materials and Methods

### Cells and reagents

The 3D/4 cells used in our study are a spontaneously-transformed line of swine macrophages purchased from ATCC (Manassas, VA) and grown in RPMI 1640 medium (Invitrogen) containing 10% fetal bovine serum (FBS). Mouse anti-ERK and anti-JNK antibodies as well as rabbit anti-phospho ERK and anti-phospho JNK antibodies (Cell Signaling), anti-cytochrome c, anti-influenza NS1, and alkaline phosphatase (AP)-conjugated anti-rabbit and anti-mouse IgG antibodies (Santa Cruz Biotechnology) were obtained from their respective providers. Anti-cleaved caspase antibody was obtained from Cell Signaling Technology, and anti-Bak antibody was obtained from EMD Chemicals. The chemicals purchased from EMD Chemicals also included inhibitors for MAP kinases, U0126 (ERK1/2), SB203580 (p38), and InSolution JNK Inhibitor II (JNK1/2), and the inhibitors for NFkB and IKK (6-Amino-4-(4-phenoxyphenylethylamino) quinazoline (Cat. 481406) and Wedelolactone (Cat. 401474), respectively).

### Virus and virus infection

A/Nanjing/108/2009 (H1N1), a pandemic (H1N1) 2009 virus, was isolated from a swab sample of an outpatient febrile child at the Nanjing Children's Hospital during the pandemic in 2009, Nanjing, China. The sampling procedure was performed in accordance with the rules set by the Institutional Review Board at the Hospital. The eight genomic segments of this virus have been fully sequenced and the raw data are deposited at Genbank under accession numbers JQ173100 through JQ173107. The virus was grown in 9-day-old embryonating chicken eggs; virus allantoic fluid (VAF) was harvested 48 hrs after inoculation, then titrated with standard haemagglutination tests (HA) and plaque assays in MDCK cells for HA and infectious viral titers, respectively [24]. For viral infection, the 3D/4 cells were trypsinized, resuspended in RPMI 1640 medium containing 10% FBS, and plated on 6-cm tissue culture plates at 5×10^6^ cells per plate 12 hrs before infection. The cells were infected with H1N1pdm inocula in VAF at a multiplicity of infection (MOI) of 1. After 1 hr of adsorption, the virus inocula were discarded and 3 ml of serum-free RPMI 1640 medium containing TPCK-trypsin (1 µg/ml, Sigma) was added. The cells were incubated at 37°C and 5% CO_2_ for various time points before cell lysates or total RNA extraction were prepared.

### Real-time RT-PCR

mRNA transcript levels of IFN-β, IL-1β, IL-6, IL-8, CCR5, IP-10, TNF-α, FasL, TRAIL, Mx, 2′5′-OAS, retinoic acid-inducible gene I (RIG-1), melanoma differentiation-associated antigen 5 (MDA-5), and glyceraldehyde-3-phosphate dehydrogenase (GAPDH) genes were analyzed by a two-step real-time RT-PCR assay as described previously [25]. 1 µg of total RNA, prepared from the cells using the RNeasy kit (Qiagen), was reverse transcribed with the QuantiTect reverse transcription kit (Qiagen) following the manufacturer's instructions. The sequences of primers used in the study are listed in Table 1. The RT reaction was carried out with the RNA after treatment with DNase I at 42°C for 2 min. Real-time PCR was conducted with 1 µl of cDNA in a total volume of 25 µl with the iQ SYBR Green Supermix (Bio-Rad) following the manufacturer's instructions. Relative expression values were normalized using an internal GAPDH control. The fold change of relative gene expression levels was calculated following the formula: 2^(ΔCt of gene−ΔCt of GAPDH)^ [25], [26]. For each reaction, melting curves were analyzed to determine the specificity of each amplicon. To determine the viral RNA level, the total RNA from infected cells was reverse transcribed and cDNA used for Taqman-based real-time PCR (Applied Biosystems) to measure viral M gene transcripts in the infected cells [27].

**Table 1:** The sequences of the primers used for detecting swine genes by realtime RT-PCR.

Gene	Primer 5′	Primer 3′
IL-1β	AGTGGAGAAGCCGATGAAGA	CATTGCACGTTTCAAGGATG
IL-6	CCTCTCCGGACAAAACTGAA	TCTGCCAGTACCTCCTTGCT
IL-8	TAGGACCAGAGCCAGGAAGA	AGCAGGAAAACTGCCAAGAA
IL-10	CTGCCTCCCACTTTCTCTTG	TCAAAGGGGCTCCCTAGTTT
CCL-5	CATGGCAGCAGTCGTCTTTA	AAGGCTTCCTCCATCCTAGC
IFN-β	AGCACTGGCTGGAATGAAAC	TCCAGGATTGTCTCCAGGTC
Mx1	CACAGAACTGCCAAGTCCAA	GCAGTACACGATCTGCTCCA
2′5′-OAS	AGCAAGGAAGCAGGAAAACA	GCTTCCCAGAAGATGCAAAG
FasL	CCCATACCCCCAAATCTTCT	CTGGACAGGGGAAGACTGAG
TRAIL	AAAGCTTTGGGCCAGAAAAT	CCAGCTCTCCATTCCTCAAG
TNF-α	CCACCAACGTTTTCCTCACT	TTGATGGCAGAGAGGAGGTT
RIG-1	ACGAAAGGGGAAGGTTGTCT	ATGCCTGCAACTTTGTACCC
MDA-5	CAGTGTGCTAGCCTGCTCTG	GCAGTGCCTTGTTTCCTCTC
GAPDH	CCACCCAGAAGACTGTGGAT	AAGCAGGGATGATGTTCTGG

### Western blot analysis

Cell lysates were prepared by lysing uninfected and infected 3D/4 cells in 1% NP-40 lysis buffer containing 1 µM PMSF, 1% aprotinin, 20 µg leupeptin ul^−1^ and 1 µM sodium vanadate (Sigma) as described previously [10]. Cell lystes were clarified by low speed centrifugation (1000 g, 5 min at 4°C) and subjected to SDS-PAGE (10 to 12%). Proteins were transferred to the Immuno-Blot PVDF membrane (Bio-Rad), and western blot analysis was performed following standard protocols [28] using rabbit or mouse anti-MAP kinase or phosphor-MAP kinase antibodies (1∶500) in TBST containing 5% fat-free milk powder for 90 mins incubation at RT. After washes, incubation with AP-conjugated anti-rabbit or anti-mouse IgG antibody for another 90 mins followed. After incubation and thorough washes, BCIP/NBT reagents (Sigma) were used for the development of colorimetric signals on the membrane. The membrane was also blotted with a monoclonal anti-actin antibody (Santa Cruz Biotechnology) for input control.

### Statistical analysis

For statistical analysis, a two-tailed Student's *t*-test was used to evaluate realtime RT-PCR data. An x^2^ analysis was used to evaluate significant differences of the data in two and more groups. The 0.05 level of probability (p<0.05) was considered statistically significant.

## Results

### 1. Susceptibility of pig macrophages to pandemic H1N1 (2009) influenza virus

**Figure 1:**
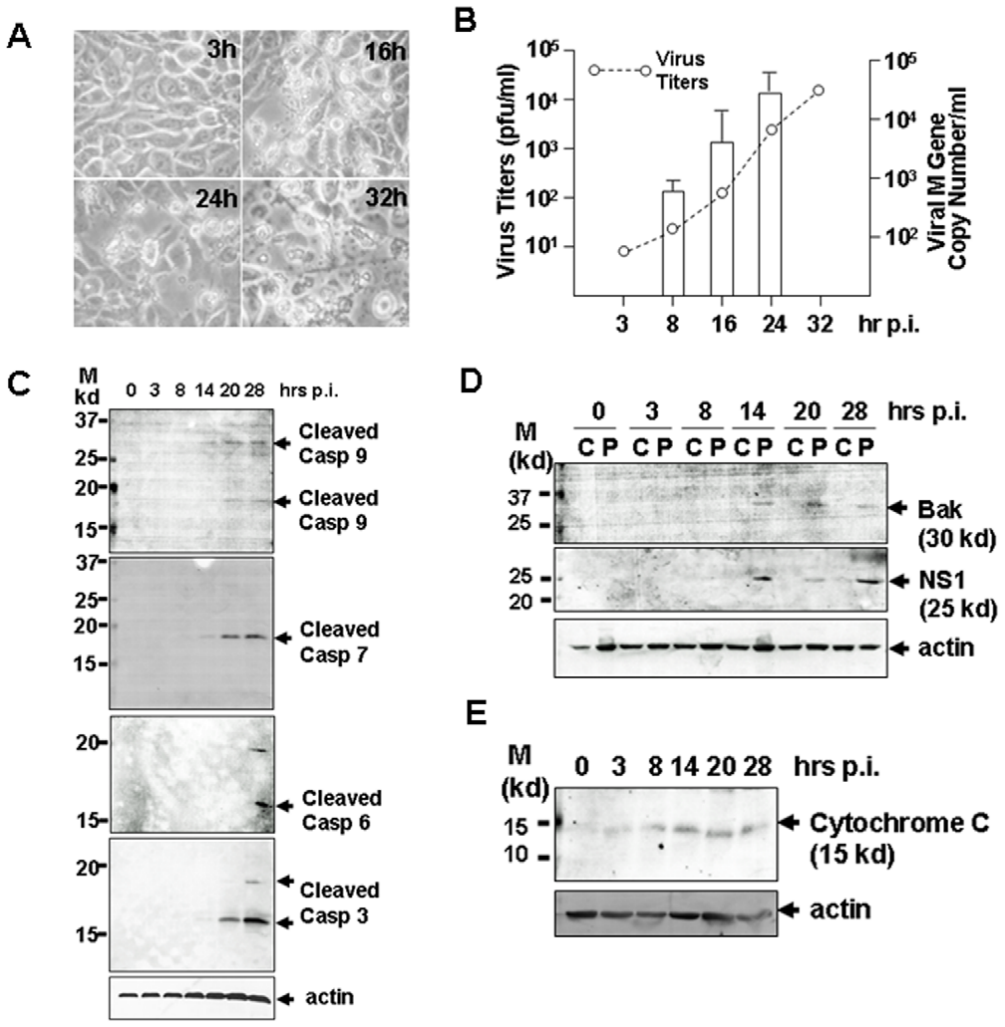
Susceptibility of swine macrophages to H1N1pdm infection. 3D/4 cells were infected with H1N1pdm at an MOI of 1 and incubated at 37°C. **A.** Cell death caused by H1N1pdm infection at 3, 16, 24, and 32 hrs post infection under a light microscope (×400). **B.** Replicative curves of H1N1pdm in 3D/4 cells. The cultural media of the infected cells were collected at various time points. Infectious viral titers were titrated in MDCK cells by a standard plaque assay. Total RNA was extracted from the infected cells and viral M gene copy numbers in the infected cells were measured with a realtime RT-PCR analysis. **C.** Activation of caspases in H1N1pdm-infected 3D/4. Cell lysates were prepared from uninfected and infected cells and subjected to SDS-PAGE and western blot analysis with anti-cleaved caspase antibodies. Cleaved caspase 3, 6, 7, and 9 were shown by western blot analyses. **D.** Detection of Bak, a Bcl-2 family member, as well as viral NS1 protein in infected cells. Cytosolic (C) and insoluble (P) fractions were prepared, respectively, for western blot analyses. **E.** Cytochrome c was released into the cytosol in infected cells. Cytosolic fractions were prepared and subjected to SDS-PAGE and western blot analysis with an anti-cytochrome c antibody.

To examine the susceptibility of pig macrophages to H1N1pdm originating from a human host, we infected 3D/4 cells with the A/Nanjing/108/2009 (H1N1). Typical cytopathic effect (CPE) appeared 16 hrs post infection and the cell monolayer was destroyed 32 hrs post infection (Fig. 1A). This result demonstrated that H1N1pdm retains the ability to infect and replicate in swine macrophages, and can reach 1.8×10^4^ PFU/ml as shown in a replicative curve (Fig. 1B). Apoptosis occurred and proceeded through the course of the infection, as we observed cleaved/activated caspase-9 as well as the emergence of downstream executioner caspases-6, -7, and -3, which eventually destroyed the infected swine macrophages (Fig. 1C). Clearly, cytochrome c was released into the cytosol (Fig. 1E), which activated mitochondria-mediated intrinsic apoptosis as early as 3 hrs post infection. Bak, a pro-apoptotic Bcl-2 family member, was upregulated as detected in the infected cells (Fig. 1D), and may be involved in the release of cytochrome c from mitochondria in swine macrophages.

### 2. Proinflammatory cytokine and TNF family responses in swine macrophages to pH1N1 infection

**Figure 2:**
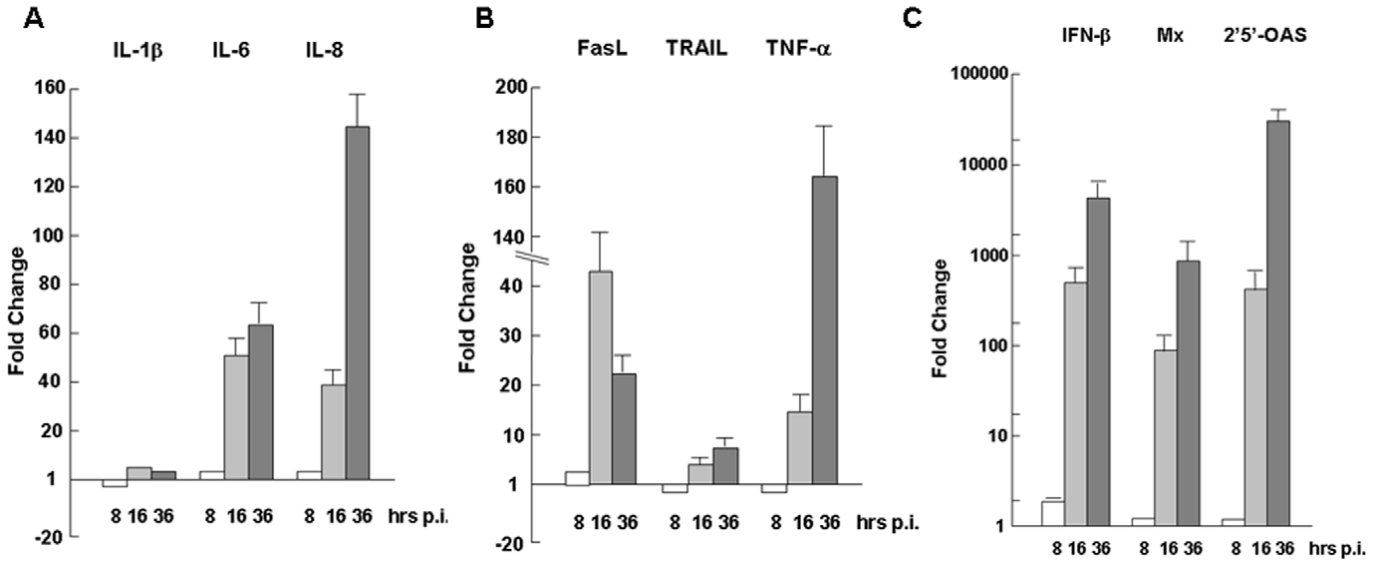
Cytokine and TNF family ligand responses to H1N1pdm infection in swine macrophages. 3D/4 cells were infected with H1N1pdm, and total RNA were prepared from infected and control cells at 8, 16, and 36 hrs post infection. After reverse transcription, cDNA was used for realtime PCR to measure the amount of gene transcripts with specific primers. Each assay was repeated at least twice. **A.** Fold changes of proinflammatory cytokine IL-1β, IL-6, and IL-8 transcripts. **B.** Fold changes of TNF family ligand FasL, TRAIL, and TNF-α transcripts. **C.** Fold change of antiviral IFN-β, and INF-inducible Mx and 2′5′-OAS transcripts.

To elucidate the pathogenesis of H1N1pdm in pigs, we examined the pattern of cytokine responses in pH1N1-infected swine macrophages. Total RNA from infected and uninfected 3D/4 cells collected at different time points post infection (p.i.) were prepared and used for realtime RT-PCR analyses with specific primers to swine cytokines. We found that the levels of proinflammatory cytokines IL-6 and IL-8 were upregulated up to 51- and 38-fold at 16 hrs, respectively, and the level of IL-8 continued to rise up to 142- fold at 36 hrs p.i.. However, the level of IL-1β remained unchanged throughout the infection (Fig. 2A), indicating that IL-6 and IL-8, as well as TNF-α (Fig. 2B) as described later, were the main proinflammatory cytokines upregulated.

We observed a robust induction of antiviral IFN-β, which rose up to 620- and 5,100-fold at 16 and 36 hrs p.i., respectively (Fig. 2C). IFN-inducible antiviral proteins Mx and 2′5.-OAS were induced accordingly up to 910- and 12,510-fold, respectively, at 36 hrs p.i. (Fig. 2C).

TNF family members were also induced in response to H1N1pdm infection, which may be attributable to cell death. We found that in pig macrophages the levels of FasL and TNF-α remained undetectable, while TNF-related apoptosis-inducing ligand (TRAIL) seemed to be most abundant before infection, based on C*t* values from realtime RT-PCR (data not shown). FasL and TNF-α were induced most robustly, but TRAIL was only mildly induced in response to infection (Fig. 2B). Among the induced, the level of TNF-α, critical in both cell death and inflammation, was sharply upregulated up to 14- and 162-fold, and FasL up to 43- and 22-fold at 16 and 36 hrs p.i., respectively. FasL and TNF-α may play a major role in H1N1pdm-triggered extrinsic apoptosis.

### 3. Activation of MAP kinases and NFkB in pH1N1-infected swine macrophages

To understand the mechanism of proinflammatory cytokine and TNF family ligand induction in H1N1pdm-infected swine macrophages, we investigated how MAP kinases were activated and whether their signaling pathways were involved in the regulation of various cytokines and TNF family ligands in pig immune cells.

**Figure 3:**
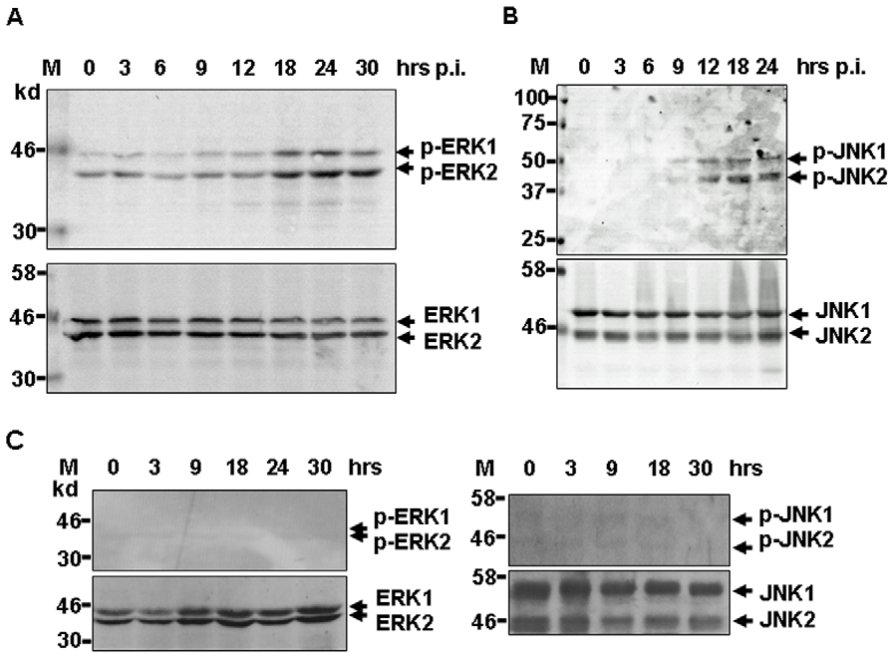
Activation of MAP kinases in H1N1pdm-infected 3D/4 cells. Swine macrophages were infected with H1N1pdm virus and cell lysates were prepared at various time points after infection, and subsequently subjected to SDS-PAGE and western blot analysis to show phosphorylation and activation of MAP kinases and NFκB. Background phosphorylation of 3D/4 cells was also measured over the time without infection. **A.** Phosphorylation of swine ERK1/2. Proteins were analyzed with anti-ERK1/2 and anti-phospho-ERK1/2 antibodies. **B.** Phosphorylation of swine JNK1/2. Proteins were analyzed with anti-JNK1/2 and anti-phospho-JNK1/2 antibodies. C. Background phosphorylation of ERK1/2 and JNK1/2. After 16 hr culture, uninfected 3D/4 cells were taken at various time points for western blot analyses to measure background phosphorylation.

**Figure 4:**
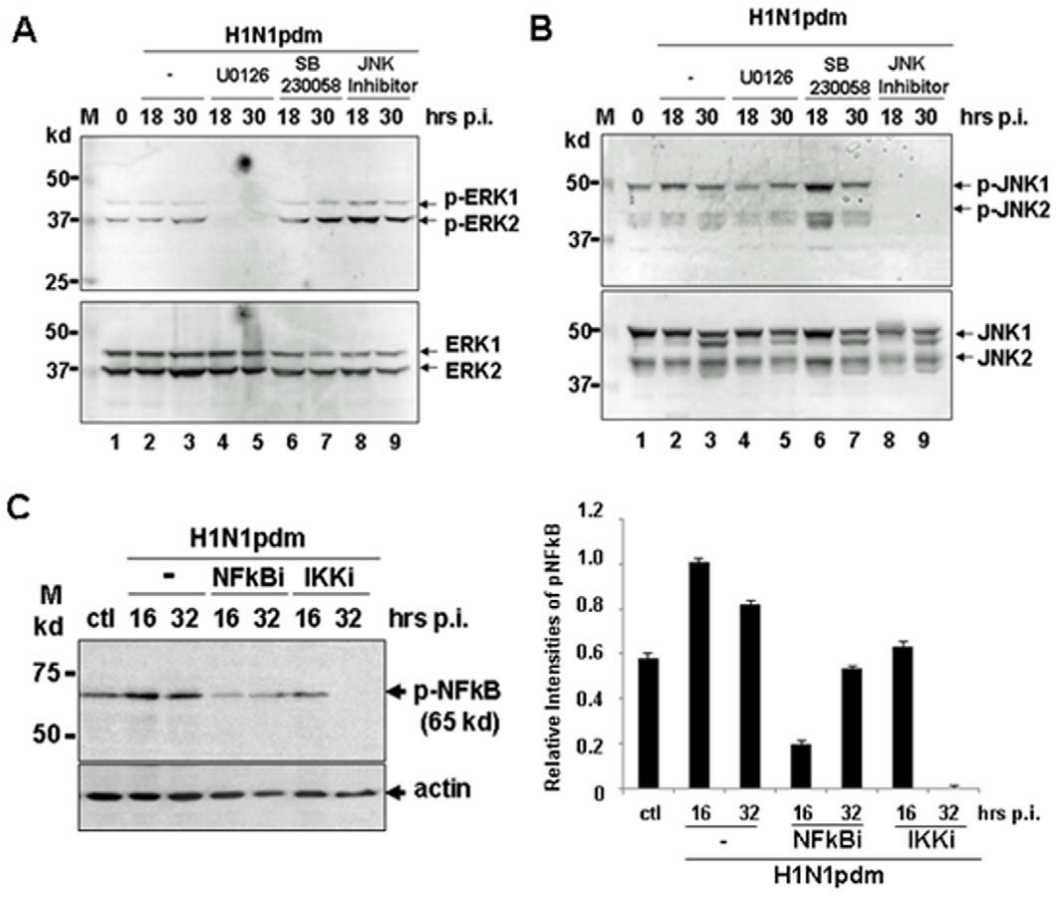
Specific inhibition of phosphorylation and activation of swine MAP kinases and NFκB in H1N1pdm-infected 3D/3 cells. Swine macrophages were pretreated with U0126, SB230058, and InSolution JNK Inhibitor, which are inhibitors of ERK1/2, p38, and JNK1/2, respectively. The final concentrations of the inhibitors were 10 µM, 5 µM, and 50 µM for U0126, SB230058, and the InSolution JNK Inhibitor, respectively. After 1 hr incubation, the treated cells were infected with H1N1pdm at an MOI of 1, and the cell lysates were prepared at 18 and 30 hrs post infection. The cell lysates were subjected to SDS-PAGE and western blot analysis with anti-phospho-ERK1/2 and anti-phospho-JNK1/2 antibodies. **A.** Specific inhibition of ERK1/2 phosphorylation and activation by U0126. **B.** Specific inhibition of JNK1/2 phosphorylation and activation by InSolution JNK inhibitor. **C.** Phosphorylation and inhibition of NFkB activation in infected 3D/4 cells. The cells were pre-treated with the inhibitors 1 hr prior to infection. The final concentrations for the NFκB and IKK inhibitors were 10 nM and 10 µM, respectively.

3D/4 cells were infected with H1N1pdm, and cell lysates were prepared at various time points for SDS-PAGE and western blot analyses with specific anti-ERK1/2 and anti-JNK1/2 antibodies. Activated forms of ERK and JNK (phospho-ERK1/2 and phosphor-JNK1/2) were detected by anti-phospho-ERK1/2 and anti-phospho-JNK antibodies. As shown in Figure 3A, ERK1/2 was basally phosphorylated at a low level before infection, but further phosphorylated between 9 and 18 hrs and thereafter p.i.. Phosphorylation and activation of JNK1/2 appeared at 9 hrs and increased to the peak around 18 hrs p.i. (Fig. 3B). Although both ERK1/2 and JNK1/2 were activated in response to H1N1pdm infection in swine macrophages, ERK1/2 remained active at basal level even before infection, so did JNK1/2 as shown in some of our experiments (Fig. 4B). However, our data showed that basal level phosphorylation of both ERK1/2 and JNK1/2 remained unchanged in uninfected 3D/4 cells through the period of our infection. In addition to ERK1/2 and JNK1/2, we have also observed the phosphorylation and activation of p38 MAP kinase in H1N1pdm-infected cells (data not shown).

### 4. Specific inhibition of the phosphorylation and activation of MAP kinases and NFkB in swine macrophages

To evaluate the role of MAP kinases in the regulation of proinflammatory cytokine responses in H1N1pdm-infected swine macrophages, we pre-treated 3D/4 cells with specific inhibitors for ERK1/2, p38, and JNK1/2 1 hr prior to infection. We then infected the cells with the virus and observed how infection-induced activation of MAP kinases was affected by inhibition of the respective MAP kinases.

As shown in Figure 4A, 3D/4 cells were pre-treated with inhibitors of ERK1/2 (U0126), p38 (SB230058), and JNK1/2 (JNK InSolution), at concentrations of 10 µM, 5 µM, and 50 µM, respectively. While the phosphorylation of ERK1/2 was unaffected by treatment with the p38 and JNK inhibitors, it was completely abolished at both 18 and 30 hrs p.i. (lines 4–5) by the ERK1/2 inhibitor U0126 (Fig. 4A). We noted that the basal level phosphorylation of ERK1/2 diminished in the presence of U0126. On the other hand, in light of the p38 and JNK inhibition with their specific inhibitors, the phosphorylation of ERK1/2 appeared to be enhanced (Fig. 4A, lines 6–9), indicating that a compensatory mechanism may exist among MAP kinases.

We observed a similar response in which a complete suppression of JNK1/2 phosphorylation was observed (lines 8–9) when the cells were pre-treated with the JNK1/2 inhibitor (Fig. 4B). However, the phosphorylation of JNK1/2 was not suppressed at all by the inhibitors of ERK1/2 and p38. We noted that there were double bands for JNK1, and a lower band of JNK1 usually appeared at a later stage of infection (30 hrs p.i.). This band was detected mainly by anti-JNK1/2, but not by anti-phospho-JNK1/2, indicating that JNK activation was transient and dephosphorylation of JNK occurred at later stages of infection, probably by an uncharacterized MAP kinase phosphatase (MKP) present in pigs.

A basal level phosphorylation of NFκB was also observed in 3D/4 pig macrophages, and was further enhanced upon H1N1pdm infection, indicating that the NFκB pathway was activated as well in infected pig macrophages (Fig. 4C). When the cells were pre-treated with specific inhibitors of NFκB (10 nM) or IKK (10 µM), the phosphorylation/activation of NFκB was effectively decreased or diminished.

MAP kinases and NFκB pathways were activated in H1N1pdm infected pig macrophages, which could be reversed or inhibited by their specific inhibitors. We used these inhibitors to study the regulation of host responses, which may be controlled by these pathways.

### 5. Distinct regulation of proinflammatory cytokines by MAP kinases

**Figure 5:**
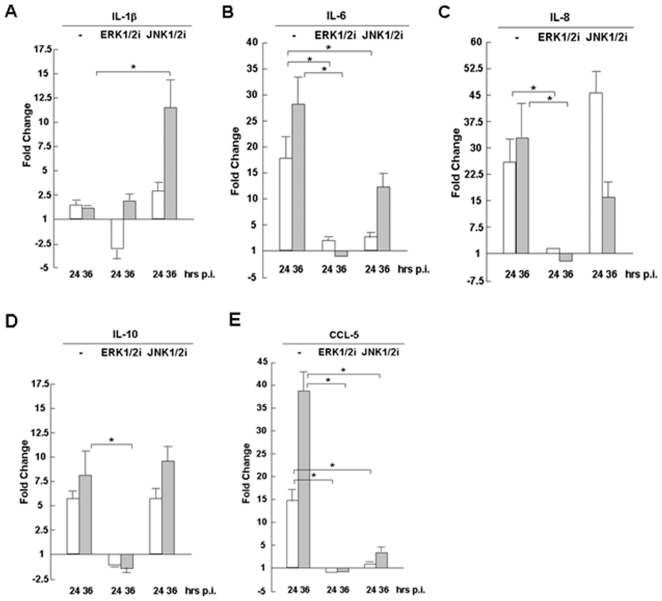
Regulation of swine proinflammatory cytokine gene transcripts by MAP kinases. 3D/4 cells were pretreated with U0126 and InSolution JNK inhibitor, which are inhibitors of ERK1/2 and JNK1/2, respectively, 1 hr before H1N1pdm infection. Total RNA was prepared at 24 and 36 hrs post infection for reverse transcription. cDNA was used for realtime PCR with specific primers to measure fold changes of cytokine transcripts at different time points. Each assay was repeated at least twice. **A–E.** Regulation of IL-1β, IL-6, IL-8, IL-10, and CCL5, respectively, by ERK1/2 and JNK1/2 inhibitors. Data show mean fold changes plus standard deviation of two or three independent assays. *p<0.05, Student's t-test.

To determine how cytokine responses are regulated by individual MAP kinases, we pre-treated the cells with ERK1/2 and JNK1/2 inhibitors, respectively, and measured the induction of the cytokines after infection with realtime RT-PCR. We observed that IL-1β was barely detected and not induced during H1N1pdm infection. Interestingly, we noticed that IL-1β was upregulated in the presence of the JNK inhibitor, although no change was observed after the treatment by the ERK inhibitor, indicating that IL-1β could have been induced in swine macrophages infected with H1N1pdm, but was virtually suppressed by JNK1/2 (Fig. 5A).

We observed that the induction of IL-6, IL-8, and IL-10 was completely suppressed in the presence of the ERK1/2 inhibitor, which indicates that IL-6, IL-8, and IL-10 inductions are all dependent on the ERK signaling pathway (Fig. 5B–D). It is interesting to note that JNK1/2 may play different roles in the induction of IL-6, IL-8, and IL-10 based on their responses in the presence of the JNK inhibitor. JNK1/2 may have moderate effects in the induction of IL-6 (Fig. 5B), but may be not relevant at all to the induction of either IL-8 or IL-10 (Fig. 5C–D).

We also noted that CCL5 (RANTES) was strongly regulated by ERK1/2 and JNK1/2 in swine immune cells. As shown in Figure 5E, induction of CCL5 was efficiently blocked in the presence of either ERK1/2 or JNK1/2 inhibitors, indicating that CCL5 is induced by H1N1pdm infection through ERK and JNK signaling pathways.

**Figure 6:**
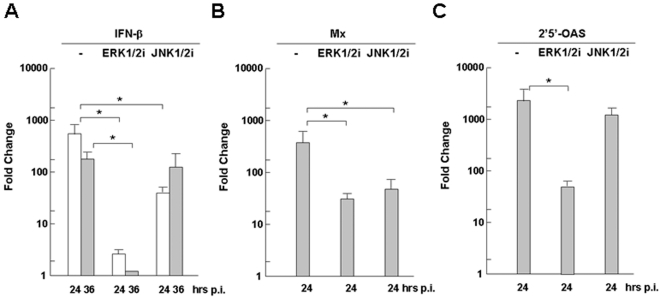
Regulation of swine IFN and antiviral gene transcripts by MAP kinases. 3D/4 cells were pre-treated with U0126 and InSolution JNK inhibitor, respectively, 1 hr before H1N1pdm infection. Total RNA was prepared at 24 and 36 hrs post infection for reverse transcription. cDNA was used for realtime PCR with specific primers to measure fold changes of cytokine transcripts at different time points. Each assay was repeated at least twice. **A–C.** Regulation of IFN-β, Mx, and 2′5′-OAS, respectively, by ERK1/2 and JNK1/2 inhibitors. Data show mean fold change plus standard deviation of two or three independent assays. *p<0.05, Student's t-test.

As for antiviral IFN-β, which was robustly induced with H1N1pdm infection in swine macrophages, ERK1/2 appeared to be essential since the induction of its mRNA transcripts was virtually abolished in the presence of the ERK inhibitor (Fig. 6A). JNK1/2 may also play a role in IFN-β induction because of its significant decrease at the earlier stage of infection (16 hrs p.i.) when 3D/4 cells were pre-treated with the JNK inhibitor. However, ERK1/2 seemed to be the primary pathway in the IFN-β induction in swine macrophages. The distinct contributions to the induction of IFN-β by ERK1/2 and JNK1/2 were also reflected in the decreased mRNA transcript levels of IFN-inducible antiviral proteins, Mx and 2′5′-OAS, in the presence of ERK and JNK inhibitors, respectively (Fig. 6B–C), which is in accordance with the suppression of the IFN-β induction by these same compounds in the infected cells. Both Mx and 2′5′-OAS were suppressed significantly by the ERK inhibitor, but only the inhibition of Mx was observed in the presence of the JNK inhibitor.

### 6. Differential regulation of TNF family ligand responses by MAP kinases

**Figure 7:**
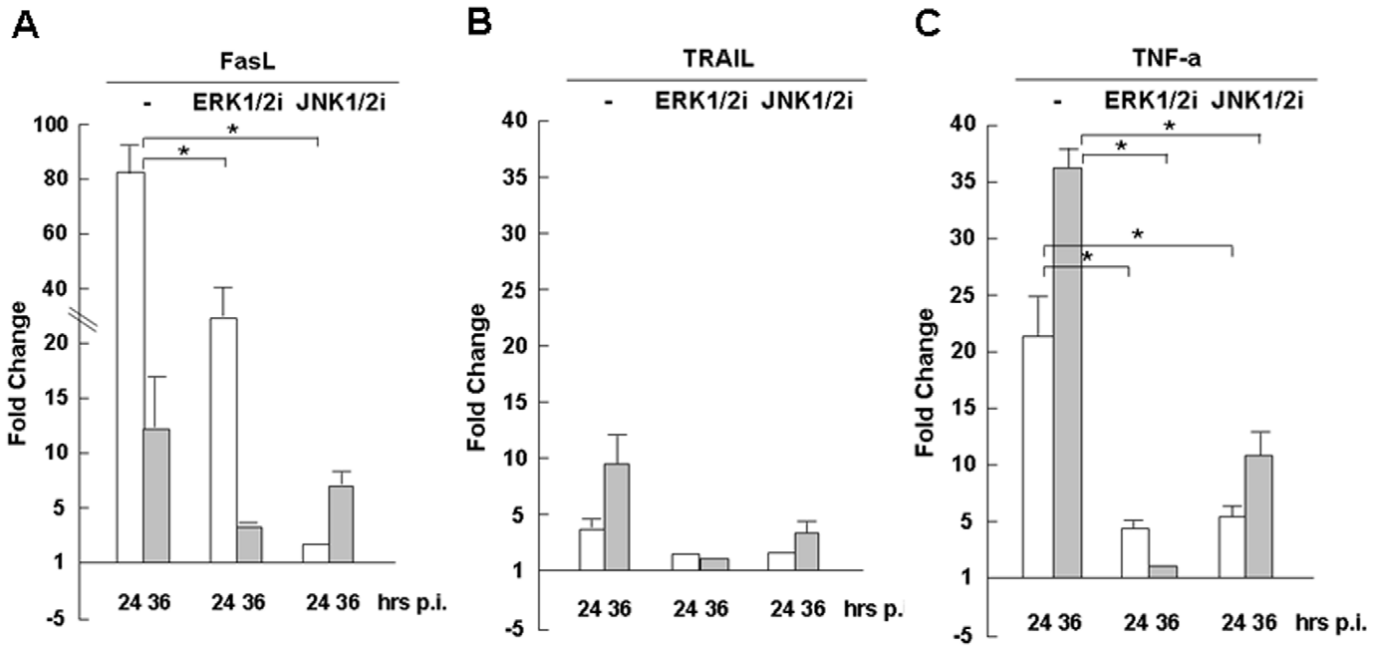
Regulation of swine TNF family responses by MAP kinases. 3D/4 cells were pretreated with U0126 and InSolution JNK inhibitor, respectively, 1 hr before H1N1pdm infection. Total RNA was prepared at 24 and 36 hrs post infection for reverse transcription. cDNA was used for realtime PCR with specific primers to measure fold changes of cytokine transcripts at different time points. Each assay was repeated at least twice. **A–C.** Regulation of FasL, TRAIL, and TNF-α, respectively, by ERK1/2 and JNK1/2 inhibitors. Data show mean fold change plus standard deviation of two or three independent assays. *p<0.05, Student's t-test.

In contrast to the abundance of TRAIL transcripts, mRNA levels of FasL and TNF-α were barely detectable by realtime RT-PCR in swine macrophages (data not shown). However, both FasL and TNF-α were induced profoundly in response to pH1N1 infection (Fig. 2B and 7A–C), while the change of TRAIL was mild. By using inhibitors, we concluded that the induction of FasL and TNF-α are mainly controlled by the ERK1/2 and JNK1/2 pathways in pig macrophages.

### 7. Cross-talk between the MAP kinase and NFκB pathways in pH1N1-infected swine macrophages

**Figure 8:**
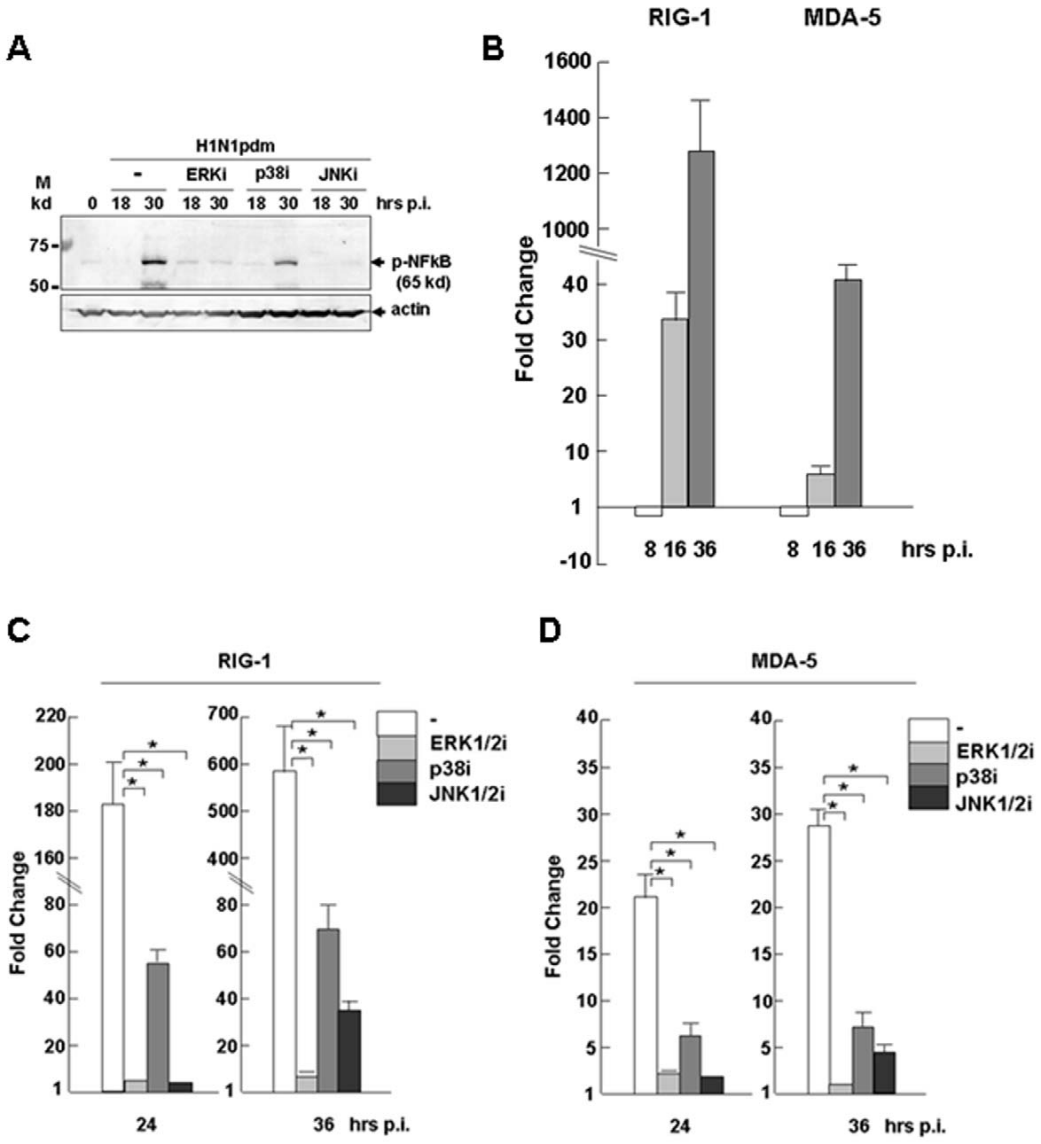
Cross-talk between MAP kinases and NFκB pathways in H1N1pdm-infected swine macrophages. **A.** Inhibition of NFκB activation in H1N1pdm-infected 3D/4 cells. The cells were pretreated with U0126, SB230058, and InSolution JNK Inhibitor, 1 hr before H1N1pdm infection. Cell lysates were prepared at 12 and 24 hrs post infection and subjected to SDS-PAGE and western blot analysis with anti-phospho-NFκB (p65) antibody. **B.** Induction of RIG-1 and MDA-5 in infected swine macrophages. Total RNA was prepared from infected cells for reverse transcription. cDNA was used for realtime PCR with RIG-1 and MDA-5 primers. **C–D.** Regulation of RIG-1 and MDA-5 induction by MAP kinases. The cells were pretreated with U0126, SB230058, and InSolution JNK inhibitor, 1 hr before H1N1pdm infection. Total RNA was prepared from infected cells for reverse transcription. cDNA was used for realtime PCR with RIG-1 and MDA-5 primers to measure fold changes of RIG-1 and MDA-5 transcripts in treated swine macrophages. Each assay was repeated at least twice. Data show mean fold change plus standard deviation of two or three independent assays. *p<0.05, Student's t-test.

The NFκB pathway could also be critical in host responses, as has been shown in humans and mice infected with influenza A virus. NFκB can be phosphorylated and activated in swine macrophages in response to H1N1pdm infection (Fig. 8A and 4C), albeit at a later stage. Interestingly, when the cells were pre-treated with ERK1/2 or JNK1/2 inhibitors, the phosphorylation of NFκB was also suppressed. However, when the cells were pre-treated with the p38 inhibitor, NFκB phosphorylation decreased much less than with ERK1/2 or JNK1/2 inhibitors (Fig. 8A). This result suggests that a cross-talk may exist between MAP kinase and NFκB pathways, and that among the MAP kinases, ERK1/2 and JNK1/2 are mainly involved.

We next examined the expression levels of RIG-1 and MDA-5, the RLR family members and cytosolic sensors for RNA viruses. We found that RIG-1 in particular was significantly induced up to 1280-fold, while MDA-5 was also upregulated up to 42-fold in infected pig macrophages (Fig. 8B). We further examined the induction of RIG-1 and MDA-5 and their relevance to MAP kinases. To do this, we pre-treated the cells with inhibitors of MAP kinases. As shown in Figure 8C, the induction of RIG-1 was completely abolished by the inhibition of ERK1/2 or JNK1/2 inhibitors, and to a much lesser extent, by the p38 inhibitor, suggesting that the induction of RIG-1 was dependent on ERK1/2 and JNK1/2, but not as much on p38. This differentially regulated pattern of RIG-1 induction by ERK1/2, p38, and JNK1/2 was similar to the suppression of NFκB phosphorylation/activation by MAP kinases (Fig. 8A), suggesting that the induction of RIG-1 was associated with ERK1/2 or JNK1/2 activation, but to a much lesser extent with p38. Since NFκB could be downstream activated by RIG-1/IPS-1 [29], [30], we postulate that ERK1/2 or JNK1/2 may activate NFκB through the activation of RIG-1/IPS-1 during H1N1pdm infection in pig macrophages.

A similar, albeit less dramatic, induction and suppression of MDA-5 expression was also observed (Fig. 8D), which indicated that MDA-5 might also be an intermediate adaptor bridging the MAP kinases ERK1/2 and JNK1/2 to the NFκB pathway activation.

## Discussion

In the present study, we have demonstrated a pattern of host responses in swine macrophages to H1N1pdm infection. Strong proinflammtory and antiviral cytokine responses including IL-6, IL-8, TNF-α, as well as IFN-β, were observed. In contrast, IL-1β was not induced, and was barely detectable in pig macrophages. This pattern differs from that in bronchoalveolar secretions of SIV-infected pigs in which IL-1β was induced but IL-8 was not [19], [20], [21], [22], [25]. The different cell types involved (macrophages and epithelial cells) may account for the difference. It has previously been reported that in human immune cells and patients a weak innate immune response, evidenced by a poor induction of proinflammatory and antiviral cytokines including IFN-β and TNF-α, has been observed in human monocyte-derived DCs and macrophages infected with H1N1pdm, compared to seasonal H1N1 infection [31]. Highly pathogenic H5N1 viral infection in human macrophages induced higher expression of IL-6 and CCL5 (RANTES) than pH1N1 [32], which may explain generally mild clinical disease among H1N1pdm-infected patients. In human macrophages, similar to our findings, IL-1β was not detected.

MAP kinase signaling pathways and their roles in the regulation of cytokines and viral replications have not been characterized in influenza-infected pig immune cells. In this study, we found that ERK1/2 and JNK1/2 could both be activated in swine macrophages. We noted that ERK1/2 was phosphorylated and active at a low level constitutively, which may be important for the rapid physiological responses required upon infection.

To elucidate the mechanism that regulates swine host responses, we used specific inhibitors of MAP kinases to pre-treat macrophages before infection. We determined that the induction of IFN-β, IL-6, IL-8, and IL-10 were regulated by ERK1/2, while JNK1/2 may only play a minor or no role in the regulation of these cytokines. As described earlier, IL-1β was not induced in response to the pH1N1 infection, which could be explained by our data indicating that its induction was in fact efficiently suppressed by JNK1/2 in swine macrophages. This may be the first time that JNK1/2 inhibitory effects on the induction of proinflammatory cytokines have been demonstrated. Previous studies found that IFN induction was dependent on the JNK1/2 signaling pathway in epithelial cells infected with influenza virus infection [33]. However, our data clearly demonstrate that ERK1/2 plays a major role in the regulation of IFN-β in pig macrophages, which may indicate that the regulation of IFN differs in different cell types. We noted that basal level activities of both ERK1/2 and JNK1/2 were constitutively present in non-infected 3D/4 cells, which may be important in the induction of proinflammatory and antiviral cytokines at the early stages of infection. Our data indicate that the induction of IL-6, IL-8, IL-10, CCL-5, as well as IFN-β, were apparent at the earliest stages of viral infection even before ERK1/2 was further activated.

We realized that a transformed monocytic cell line, instead of primary cells, was used in the study, which may compromise the significance of our data. Basal level phosphorylation of both ERK1/2 and JNK1/2, which may affect certain cytokine production, would be minimal in primary monocytes. However, specific inhibitors used in the study completely wiped out phosphorylation of both ERK1/2 and JNK1/2 (Fig. 4). The effect of MAP kinase phosphorylation and activation on the regulation of affected cytokines as observed in our study with the inhibitors is, therefore, valid, even though the cells were not primary cultures.

Macrophages appear to die inevitably of apoptosis when infected with influenza virus [26]. The Fas-mediated extrinsic apoptotic pathway is apparently triggered by TNF family ligands. While both FasL and TNF-α were induced vigorously upon the viral infection, induction of TRAIL was rather mild in H1N1pdm-infected swine macrophages. We knew previously that FasL and TNF-α were barely detectable, while the level of TRAIL remained high prior to the infection based on our realtime RT-PCR data (Ct) (Xing et al., unpublished data). We can therefore presume that H1N1pdm-induced apoptosis may be mainly attributed to FasL and TNF-α, while pig macrophages could be resistant to TRAIL, since the cells remained intact despite the presence of a high level of TRAIL before infection. Furthermore, we were also able to determine that both ERK1/2 and JNK1/2 were involved in the induction of FasL, TNF-α, and TRAIL. FasL is also regulated by ERK1 in chicken macrophages infected with an H9N2 avian influenza virus [34].

Both toll-like receptors (TLR) and RNA helicases, such as RIG-1 and MDA-5, are critical to antiviral innate immunity [35], [36]. As a cytosolic sensor, RIG-1 binds to dsRNA and viral ssRNA that contain a 5′-triphosphate not present in host RNA, and then is recruited to mitochondrial protein IPS via the CARD domain, leading to activation of NFκB, IRF-3/-7, and induction of IFN [37], [38], [39]. RIG-1 can be induced by viral infection [40]. In this study, we observed a robust induction of RIG-1 and MDA-5 in H1N1pdm-infected swine macrophages, which appeared to be suppressed completely by inhibitors of ERK1/2 or JNK1/2, but to be a much lesser extent, by the inhibitor of p38. This indicates that the induction of RIG-1 or MDA-5 depends on the activation of ERK1/2 and JNK1/2 in pig macrophages. We postulate a mechanism, therefore, that the cross-talk between MAP kinase and NFκB pathways is through the regulation of RIG-1 and maybe MDA-5, and that ERK1/2 controls the activation of NFκB, leading to the induction of IFN in swine macrophages.
